# Effect of chronic ethanol exposure on the liver of *Clock-*mutant mice

**DOI:** 10.1186/1740-3391-7-4

**Published:** 2009-04-01

**Authors:** Takashi Kudo, Toru Tamagawa, Shigenobu Shibata

**Affiliations:** 1 Department of Pharmacology, School of Advanced Science and Engineering, Waseda University, Wakamatsu-cho 2-2, Shinjuku-ku, Tokyo, 162-8480, Japan

## Abstract

In humans, chronic ethanol consumption leads to a characteristic set of changes to the metabolism of lipids in the liver that is referred to as an "alcoholic fatty liver (AFL)". In severe cases, these metabolic changes result in the enlargement and fibrillization of the liver and are considered risk factors for cirrhosis and liver cancer. *Clock*-mutant mice have been shown to display abnormal lipid metabolism and alcohol preferences. To further understand the potential interactions between ethanol consumption, lipid metabolism, and the circadian clock, we investigated the effect of chronic ethanol intake on the lipid metabolism of *Clock*-mutant mice. We found that ethanol treatment produced a number of changes in the liver of *Clock*-mutant mice without impacting the wild-type controls. First, we found that 8 weeks of exposure to ethanol in the drinking water increased the weight of the liver in *Clock*-mutant mice. Ethanol treatment also increased triglyceride content of liver in *Clock*-mutant and wild-type mice. This increase was larger in the mutant mice. Finally, ethanol treatment altered the expression of a number of genes related to lipid metabolism in the *Clock*-mutant mice. Interestingly, this treatment did not impact circadian clock gene expression in the liver of either genotype. Thus, ethanol produces a number of changes in the liver of *Clock*-mutant mice that are not seen in the wild-type mice. These changes are consistent with the possibility that disturbance of circadian rhythmicity associated with the *Clock *mutation could be a risk factor for the development of an alcoholic fatty liver.

## Background

In humans, chronic ethanol consumption leads to a characteristic set of changes to the metabolism of lipids in the liver that is referred to as an "alcoholic fatty liver" (AFL) [1]. This condition is characterized by an increase in liver weight [2], the accumulation of triglycerides and changes in expression of genes involved in lipid metabolism [3]. In severe cases, these changes eventually lead to inflammation [4] and steatohepatitis [5] and are considered risk factors for cirrhosis and liver cancer in humans. Most of these changes are also observed in rodents [6] allowing us to use chronic alcohol exposure in rodents as a model to understand the human condition.

In recent years, many of the genes responsible for the generation of circadian rhythms have been identified [7]. Many of these genes have been found to be expressed in the liver where the transcription of a number of key genes is regulated on a circadian time scale [8]. Mice containing a mutation in the transcription factor, CLOCK, are unable to generate circadian rhythms in behavior and hepatic gene expression [9,10]. Interestingly, recent studies suggest that *Clock*-mutant mice exhibit abnormal triglycerides [11], cholesterol metabolism [12], and become obese [13]. Therefore, to understand the potential interactions between ethanol consumption, lipid metabolism and the circadian clock, we investigated the effect of chronic ethanol intake on the lipid metabolism of *Clock*-mutant mice.

## Methods

### Animals

*Clock*-mutant mice were purchased from The Jackson Laboratory (stock no. 002923, Bar Harbor, ME). These mice, originally with the *Clock *allele on a C57BL/6J background, were backcrossed using a Jcl: ICR background more than eight generations. We placed them in the ICR genetic background to enhance the robustness of the breeding and care of the young. Other groups have reported that the *Clock*-mutant mice with a C57BL/6J background died in their infancy because the dams did not care for them [14-16]. *Clock*-mutant mice were heterozygous, and genotypes were determined using PCR. We used eight- to ten-week-old female wild-type and *Clock*-mutant mice that were isogenic siblings born in the same litters. Female mice were used because females are more vulnerable than males to the effects of alcohol [17]. Mice were maintained on a light-dark (LD) cycle (12 hours light, 12 hours dark with lights on at 8:00 a.m., room temperature of 23 ± 1°C) and provided with a standard diet (Oriental Yeast Co., Ltd., Tokyo, Japan) and water *ad libitum*. During the ethanol treatment, wild-type and *Clock*-mutant mice were given water or 15% ethanol for 8 weeks (n = 16 per group), and then mice were sacrificed at ZT 0, 6, 12, or 18. Zeitgeber time (ZT) 0 and 12 were the lights-on and lights-off times, respectively. Animal care and experiments were conducted under the permission of the 'Experimental Animal Welfare Committee in the School of Science and Engineering at Waseda University' (permission number: 05G19).

### Assay for serum triglyceride, cholesterol, free fatty acid, alanine aminotransferase, aspartate aminotransferase, and ethanol content

Blood samples (500 to 750 μl) from each mouse were collected from the orbital sinus and centrifuged. A sample (20 μl) of the serum from each mouse was used to obtain triglyceride content with the Triglyceride E-test Wako (Wako Pure Chemical Industries, Osaka, Japan), cholesterol content with the cholesterol E-test Wako (Wako Pure Chemical Industries, Osaka, Japan), free fatty acid content with the NEFA C-test Wako (Wako Pure Chemical Industries, Osaka, Japan), alanine aminotransferase, aspartate aminotransferase content with the Transaminase CII-test Wako (Wako Pure Chemical Industries, Osaka, Japan), and ethanol content with the F-kit Ethanol UV-test UV method (r-biophram, Darmstadt, Germany). These were done according to the instructive manual.

### Assay for liver triglyceride content

An assay for liver triglyceride content was performed based on methods described by Yokode *et al*. [18]. From each mouse, 0.2 g of liver tissue was homogenized in a Polytron homogenizer with 4 ml chloroform/methanol (2/1, v/v), after which 0.8 ml of 50 mM NaCl was added. A sample (50 μl) of the organic phase was mixed with 7.5 mg of Triton X-100. After evaporation of the organic solvents, the lipid in the detergent phase was used to measure TG content with the Triglyceride E-test Wako (Wako Pure Chemical Industries, Osaka, Japan).

### Oil red O stain of the liver

In order to elucidate whether or not ethanol led to higher lipid accumulation in the liver of *Clock*-mutant mice compared to wild-type mice, we examined the accumulation of lipids using the oil red O stain method [11]. Mouse livers were fixed with 10% formalin and sliced in 10 μm sections with a cryostat (LEICA, Wetzlar, Germany). Next, the livers were washed in PBS solution for 30 sec, 60% isopropyl alcohol for 1 min, and stained by oil red O for 10 min at 37°C. Thereafter, slices were fractionated with 60% isopropyl alcohol for 2 min, washed with PBS solution for 2 min, and stained with hematoxylin for 5 min. After the 2 min wash in PBS solution, slices were colored with lithium carbonate for 30 sec, washed with PBS solution for 5 min, and cover slipped.

### RNA isolation and Real time RT-PCR

Total RNA was extracted using ISOGEN Reagent (Nippon Gene, Tokyo, Japan). Fifty nanograms of total RNA was reverse transcribed and amplified using the One-Step SYBR RT-PCR Kit (TaKaRa, Otsu, Japan) in the iCycler (BIO RAD, Hercules, CA). Specific primer pairs were designed based on the following published data on the *Acc1*, *β-actin*, *Dbp*, *Mtp*, and *Per2 *genes in GenBank (Table 1). RT-PCR was executed under the following conditions: cDNA synthesis at 42°C for 15 min followed by 95°C for 2 min, PCR amplification for 40 cycles with denaturation at 95°C for 5 sec, and annealing and extension at 60°C for 20 sec. The relative light unit of the target gene PCR products was normalized to that of *β-actin*. A melt curve analysis was then performed.

**Table 1:** Sequences of primer pairs used to amplify each PCR product

Gene	Sequence	Predicted Size (bp)	GenBank Accession No.
*Acc1*			
Sense	5'-GCACTCCCGATTCATAATTG-3'	141	NM_133360 (34–185)
Antisense	5'-CCCAAATCAGAAAGTGTATC-3'		
*Aco*			
Sense	5'-ATCTATGACCAGGTTCAGTCGGGG-3'	237	NM_015729 (1312–1548)
Antisense	5'-CCACGCCACTTCCTTGCTCTTC-3'		
*β-actin*			
Sense	5'-TGACAGGATGCAGAAGGAGA-3'	131	AK075973 (1009–1139)
Antisense	5'-GCTGGAAGGTGGACAGTGAG-3'		
*Dbp*			
Sense	5'-CCGTGGAGGTGCTAATGACCT-3'	105	NM_016974 (984–1087)
Antisense	5'-CCTCTGAGAAGCGGTGTCT-3'		
*Mtp*			
Sense	5'-GCCCTAGTCAGGAAGCTGTG-3'	127	NM_008642 (1300–1426)
Antisense	5'-CCAGCAGGTACATTGTGGTG-3'		
*Per2*			
Sense	5'-TGTGTGCTTACACGGGTGTCCTA-3'	142	AF036893 (5563–5704)
Antisense	5'-ACGTTTGGTTTGCGCATGAA-3'		
*Ppar α*			
Sense	5'-TCTTCACGATGCTGTCCTCCT-3'	142	NM_011144 (1395–1475)
Antisense	5'-GGAACTCGCGTGTGATAAAGC-3'		

### Statistical Analysis

The values are expressed as means ± standard error of the mean. For statistical analysis, one-way or two-way ANOVA were applied and post hoc analysis was conducted in the Fisher PLSD test.

## Results

### Chronic ethanol increased liver weight in *Clock*-mutant mice

**Figure 1:**
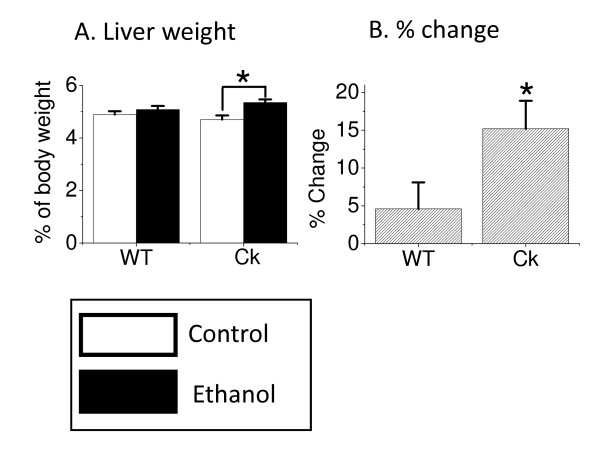
**Chronic ethanol exposure increases liver weight in *Clock*-mutant mice**. Eight- to ten-week-old wild-type (WT, n = 16) and *Clock*-mutant (Ck, n = 16) mice were given 15% ethanol in drinking water for 8 weeks. At the end of this treatment, liver and body weights were measured. *Clock*-mutant mice exhibited a significant (ANOVA, Fisher PLSD, *P *< 0.05) increase in liver/body weight (A) as well as % change in liver/body weight (B).

Chronic alcohol consumption leads to hypertrophy of the liver in humans [2] and rats [19]. Therefore, we sought to determine the impact of exposure to ethanol (8 weeks) on the mouse liver. We found that this ethanol treatment significantly increased the liver weight of the *Clock*-mutant mice (Fisher PLSD, F(3, 60) = 3.894, *P *< 0.05, Fig. 1) while wild-type mice did not exhibit a change in liver weight. There were no differences between wild-type mice and *Clock*-mutant mice in the ethanol intake or in serum levels of ethanol (Table 2). Similarly, there were no differences in food intake and body weight between the two genotypes (Table 2). Finally, alanine aminotransferase (ALT) and aspartate aminotransferase (AST) are important enzymes produced by the liver and serum levels of these enzymes are widely used biomarkers of liver health [20]. We found that ethanol treatment did not alter levels of AST or ALT in either genotype (Table 2). These results demonstrate that the livers of *Clock*-mutant mice exhibit a weight increase under conditions of ethanol exposure that do not impact wild-type mice.

**Table 2:** Impact of ethanol exposure on wild-type and *Clock*-mutant mice

	Wild-type		*Clock*-mutant	
	Control	Ethanol	Control	Ethanol
Ethanol Intake (g/kg/day)	na	30.5 ± 2.0	na	27.2 ± 1.6
Food Intake (g/kg/day)	154 ± 11	136 ± 13	154 ± 14	136 ± 10
Body Weight (g)	32.3 ± 0.6	32.3 ± 0.6	33.0 ± 0.5	31.9 ± 0.5
Serum Ethanol (mM)	na	9.0 ± 2.3	na	9.4 ± 1.8
Serum TG (mg/dl)	68 ± 6.4	84 ± 8.5	62 ± 4.8	71 ± 4.9
Serum CH (mg/dl)	64.2 ± 4.7	60.2 ± 4.8	64.5 ± 3.0	62.2 ± 2.9
Serum FFA (mEq/l)	0.50 ± 0.03	0.53 ± 0.03	0.52 ± 0.03	0.46 ± 0.02
Serum ALT (Karmen)	28.0 ± 0.7	26.1 ± 0.5	30.1 ± 1.3	27.2 ± 0.3
Serum AST (Karmen)	75.0 ± 6.5	61.8 ± 2.9	62.8 ± 4.2	59.6 ± 2.3

TG, triglycerides; CH, cholesterol; FFA, free fatty acids; ALT, alanine aminotransferase; AST, aspartate aminotransferase, na; not available

### Chronic ethanol increased liver triglyceride content in *Clock*-mutant mice

**Figure 2:**
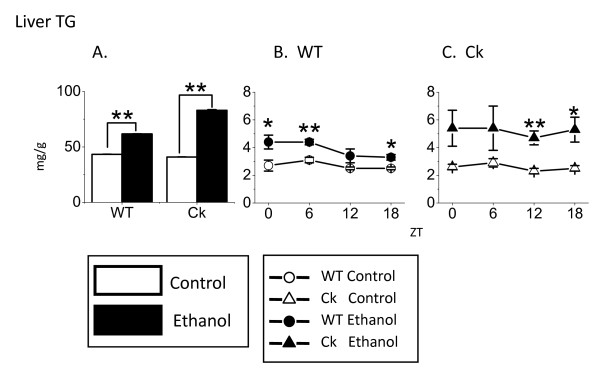
**Chronic ethanol increases liver triglycerides (TG) content in *Clock*-mutant mice**. (A) Ethanol exposure significantly increased the TG levels in both WT (n = 16) and Ck (n = 16) mice. Further analysis by two-way ANOVA indicates that the ethanol-induced increase was greater in the mutant mice (*P *< 0.01). (B) In WT mice, ethanol exposure significantly increased liver TG at ZT 0, 6, and 18 (n = 4 for each time point). (C) In Ck mice, ethanol exposure significantly increased liver TG at ZT 12 and 18 (n = 4 for each time point). Values are means ± SEM. Significance was initially determined using the one-way ANOVA followed by Fischer PLSD (*P < 0.05, **P < 0.01).

**Figure 3:**
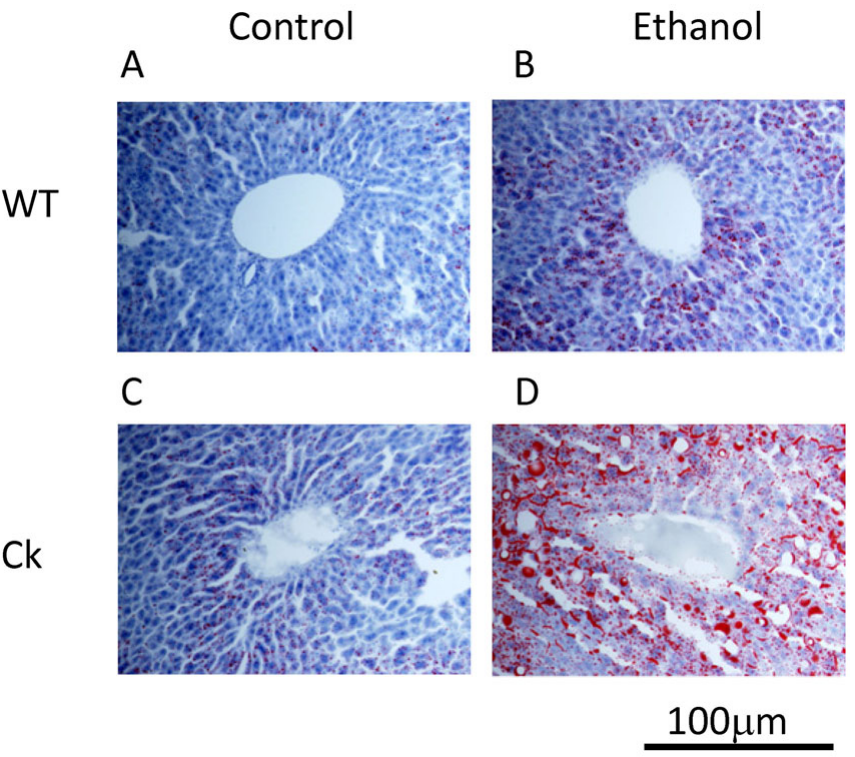
**Chronic ethanol increases liver lipid content in *Clock*-mutant mice**. The intensity of staining of liver tissue with oil red O provides a qualitative measure of the lipid content. We found that ethanol exposure increases the intensity of the oil red O staining. Representative oil red O staining of liver tissue in WT and Ck mice on control and ethanol (A-D). Images from the stained liver were magnified ×400 and scale bar is shown.

Alcohol consumption can cause an increase in liver triglyceride content in humans [3] and rats [21]. Therefore, we also measured the impact of ethanol exposure on mouse liver triglyceride levels. We found that ethanol intake increased triglyceride content in the liver of both genotypes (Fisher PLSD, F(3, 60) = 18.0, *P *< 0.01, Fig. 2A). Moreover, the ethanol-induced increase in liver triglyceride content was significantly higher in *Clock*-mutant mice compared with wild-type (Fisher PLSD, F(3, 60) = 18.0, *P *< 0.01). We measured liver triglyceride throughout the daily cycle (ZT 0, 6, 12, and 18) and found that the ethanol treatment increased triglyceride levels regardless of the time of day (Fig. 2B and 2C). Histological analysis indicated that the liver tissue from the ethanol treated mice exhibited greater staining by oil red O. While qualitative, this increase in staining is consistent with lipid accumulation in ethanol treated mice (Fig. 3). In contrast to the data from the liver, we found that ethanol exposure did not alter measured levels of triglyceride or cholesterol in the serum of mice from either genotype (Table 2). This data indicates that ethanol treatment increases levels of triglyceride in the mouse liver and that this lipid accumulation is greater in *Clock*-mutant mice than in wild-type mice.

### Chronic ethanol altered the expression of genes involved in lipid metabolism in *Clock*-mutant mice

**Figure 4:**
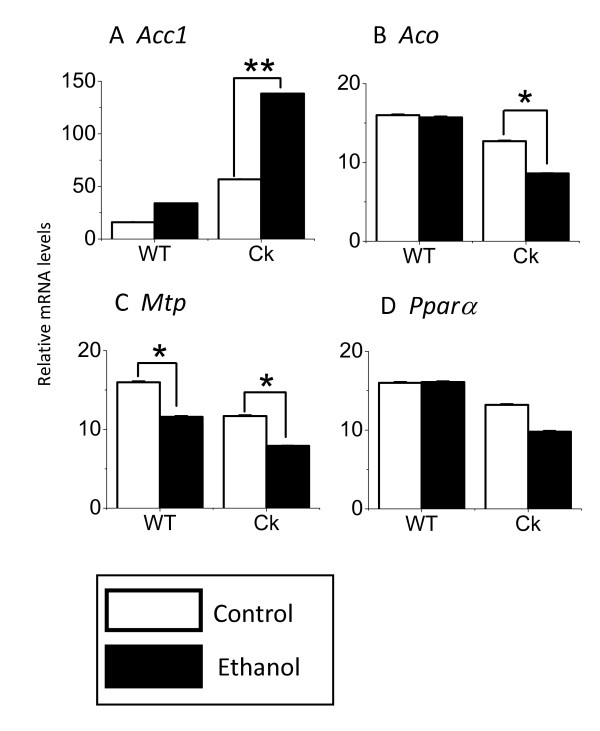
**Chronic ethanol alters the expression of genes involved in lipid metabolism in *Clock*-mutant mice**. We investigated the impact of ethanol treatment on 4 genes that are critically involved in lipid metabolism. Expression of *Acc1 *(A), *Aco *(B), *Mtp *(C), and *Pparα *(D) were measured from the liver (n = 16) with RT-PCR and product normalized to that of *β-actin*. Significance was determined using the one-way ANOVA followed by Fischer PLSD (*P < 0.05, **P < 0.01).

In order to investigate the possible mechanisms underlying these effects of ethanol on the lipid content, we investigated the impact of ethanol treatment on 4 genes that are critically involved in lipid metabolism (Fig. 4). First, we found that ethanol treatment significantly increased the expression of *Acc1 *in the liver of *Clock*-mutant (Fisher PLSD, F(3, 60) = 13.069, *P *< 0.01) but not wild-type mice. Second, we found that ethanol treatment decreased the expression of *Aco *(F(3, 60) = 6.27, *P *< 0.05) in *Clock*-mutant but not wild-type mice. Third, we found that both *Clock*-mutant and wild-type mice exhibited an ethanol-induced reduction in *Mtp *expression (Fisher PLSD, F(3, 60) = 7.601, *Clock*-mutant: *P *< 0.05; wild-type: *P *< 0.05). Finally, we found that ethanol treatment tended to decrease the expression of *Pparα *in *Clock*-mutant but this effect was not significant. These data demonstrate that ethanol-treatment can alter the expression of genes involved in lipid metabolism in the mouse liver and that *Clock*-mutant mice exhibit different ethanol-induced change than wild-type mice.

### Chronic ethanol does not alter the expression of circadian clock genes

**Figure 5:**
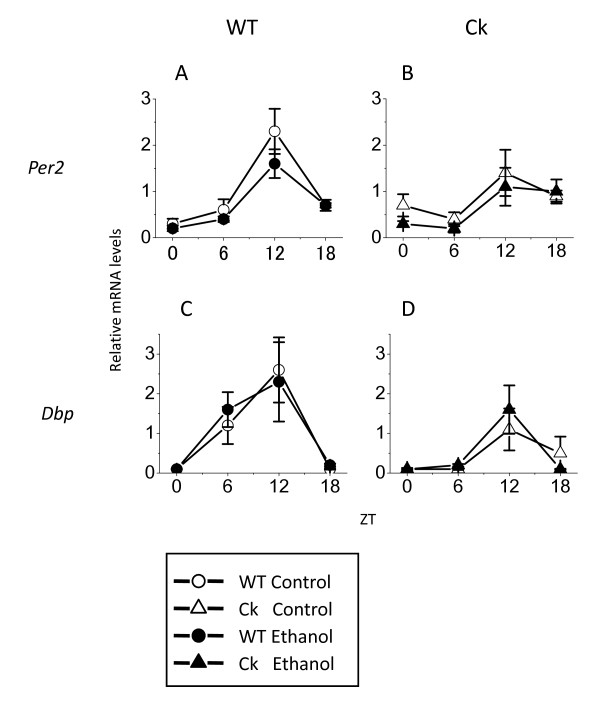
**Chronic ethanol does not alter the expression of circadian clock genes**. After exposure to ethanol, mouse livers were collected every 6 hours (n = 4 per time point). Expression of *Per2 *(A, B) and *Dbp *(C, D) was measured from the liver with RT-PCR and product normalized to that of *β-actin*. Values are means ± SEM and normalized as average levels of water-received wild-type mice become 1. Significance was determined using the one-way ANOVA followed by Fischer PLSD.

Finally, we examined the impact of ethanol on the expression of circadian clock genes in the mouse liver. In wild-type mice, the expression of both *Per2 *and *Dbp *showed a clear daily rhythm with peaks at ZT 12. In *Clock*-mutant mice, the amplitude was damped (*Per2*, control: from 7.7 to 3.5, ethanol: 8.0 to 5.5, *Dbp*, control from 26 to 11, ethanol from 23 to 16) but still exhibited peak expression at ZT 12. Exposure to ethanol did not significantly affect the average level of *Per2 *and *Dbp *gene expression in either genotype (Fig. 5). Therefore, our data suggests that ethanol exposure does not alter the expression of circadian clock genes in the liver.

## Discussion

The liver plays a key role in the metabolism of alcohol and is also sensitive to the deleterious effects of chronic alcohol consumption. In humans, chronic alcohol consumption leads to a characteristic set of changes to the metabolism of lipids in the liver that is referred to as an "alcoholic fatty liver". In humans, AFL is characterized by an increase in liver weight, accumulation of triglyceride and changes in the expression of genes involved in lipid metabolism. In severe cases, these metabolic changes result in the enlargement and fibrillization of the liver and are considered risk factors for cirrhosis and liver cancer.

Previous work suggests that there may be links between mutations in the circadian system and liver metabolism. The transcription of many genes in liver, including those involved in lipid metabolism, is regulated on the circadian time scale [8,22]. Furthermore, mice with mutations in core elements of the circadian timing system also exhibit deficits in liver function. In the best studied example, the *Clock*-mutant mice [9,23] exhibit abnormal triglyceride [11], abnormal cholesterol metabolism [12], and become obese over time [13]. In humans, common genetic variations in CLOCK transcription factor are associated with fatty liver disease [24]. To further understand the potential interactions between ethanol consumption, lipid metabolism, and the circadian clock, we investigated the effect of chronic ethanol intake on the lipid metabolism of *Clock*-mutant mice. We used a previously established protocol in which mice are given drinking water with 15% ethanol over the course of 8 weeks [25].

We found that ethanol exposure increased the weight of the liver in *Clock*-mutant, but not wild-type mice (Fig. 1). Previous studies have found that alcohol consumption leads to increase in weight of liver of humans [2] and rats [19]. This difference in liver weight between the two genotypes could not be obviously explained by consumption. The body weight between the two genotypes was not different with or without ethanol exposure (Table 2). Finally, we saw little evidence for liver damage in the mice exposed to this treatment regime. ALT and AST are important enzymes produced by the liver and serum levels of these enzymes are widely used biomarkers of liver health [20]. Alcohol consumption leads to increase in ALT and AST of humans [26] and rats [27], but not in wild-type or *Clock*-mutant mice in the present study (Table 2). Thus, the 8-week exposure to ethanol caused a modest and selective increase in the weight of the liver of *Clock*-mutant mice without causing overt liver damage. The mechanisms underlying toxicity of ethanol associated with clock gene expression should be discussed from the view point of pharmacokinetics as well as pharmacodynamics. The circadian rhythm of alcohol dehydrogenase activity was previously reported in the rat liver [28]. Therefore, alcohol metabolism might be disturbed in *Clock*-mutant mice, and resulted in increase of blood ethanol level. However, ethanol intake, and importantly, serum levels of ethanol were not different between the wild-type and *Clock*-mutant mice (Table 2). The hypothermic, hypnotic, and also lethal effect of ethanol showed circadian variations [29], suggesting that there is circadian rhythm in pharmacodynamics of ethanol. Taken all evidences together, it is suggested that we should consider the ethanol toxic effect in the circadian rhythms of the pharmacokinetics and pharmacodynamics, although mice did not show any evidence for a daily rhythm in the levels of liver triglyceride.

Triglyceride is one of the most important lipids in the liver and triglyceride in liver and serum are widely used measures of lipid metabolism [30]. Ethanol treatment also increased triglyceride content of liver in *Clock*-mutant mice as well as wild-type controls (Fig. 2). The ethanol-induced increase in triglycerides was larger in the mutants than in the wild-type mice. Previous studies in humans [31] and rats [32] have also reported that ethanol increases levels of triglyceride. There were the circadian rhythm in *Aco *and *Pparα*, but not in *Acc1 *and *Mtp*. Analysis of oil red O staining provides a qualitative measure of the lipid content in the liver as higher levels of lipids leads to stronger staining [11]. We found again that the ethanol-treated group exhibited stronger staining than untreated and that this increase in staining was more robust in the *Clock-*mutant mice (Fig. 3). Therefore, both the triglyceride measurements and the histological analysis suggest that ethanol increases the lipid content in the liver and that this effect is more robust in the *Clock-*mutant mice.

CLOCK is a transcription factor and *Clock*-mutant mice exhibit a number of changes in the transcription of gene in the liver [33]. The ethanol-induced increase in lipid content of the liver of *Clock*-mutant mice could be explained by changes in triglyceride synthesis, degradation, or transport from the liver to blood. Therefore, we used RT-PCR methods to measure the expression of some of the genes involved in triglyceride synthesis, degradation, or transport (Fig. 4). Triglyceride is synthesized by fatty acids and glycerol released by the glycolytic pathway [34]. In triglyceride synthesis, ACC1 [1] is the rate limiting enzyme of Acyl-CoA synthesis from fatty acid in mouse. We found that the *Clock*-mutant mice had higher baseline levels of *Acc1 *and that ethanol-exposure produced a strong increase in the expression of this gene. Another important player in this pathway is *Aco*, which is involved in triglyceride degradation by peroxisome β-oxidation [35]. In the present experiment, gene expression of *Aco *in the liver of *Clock*-mutant mice was significantly reduced by ethanol intake. Thus, a reduction of β-oxidation by the *Clock *mutation could also contribute to the high liver triglyceride content. Finally, *Mtp *is a gene involved in the triglyceride transport from the liver to blood [36]. Ethanol-exposure decreases expression of *Mtp *in the liver of *Clock*-mutant mice. A reduction of triglyceride export from the liver through reduced *Mtp *expression may also explain the increase of triglyceride content in the liver of *Clock*-mutant mice. This gene expression data suggests that exposure of *Clock*-mutant mice to ethanol produces a coordinated set of changes in the expression of genes involved in triglyceride synthesis, degradation and transport.

We also found that the expression of *Pparα*, which is clock-controlled gene, was reduced by ethanol intake in the *Clock-*mutant, but not in wild-type mice. It has been reported that *Pparα *knock-out mice receiving ethanol for eight weeks developed severe fatty liver [37]. Therefore, the ethanol-induced decrease in *Pparα *expression could be responsible for the abnormal lipid accumulations in the liver of *Clock*-mutant mice. Interestingly, both *Aco *and *Mtp *genes have PPRE sequences in their promoter regions and receive a transcriptional regulation by *Pparα *[38,39]. Thus, ethanol-induced reduction in *Pparα *could explain the reduction in the transcription of *Aco*, and *Mtp*. The findings that the expression of these genes were reduced in untreated *Clock*-mutant mice in comparison with wild-type mice suggests that CLOCK is important for maintaining higher expression levels of these metabolic genes. In other words, these metabolic genes are controlled by *Clock *genes through PPARα. The *Clock *knock-out mouse has no central circadian phenotype [40], but *Clock *gene seems to be indispensible for the functioning of peripheral tissue clocks [41]. CLOCK protein has also been shown to be a histon acetyl-transferase and this gene-regulatory function[42], which is not necessarily only acting on other clock genes like *Bmal1*, may have to do with the liver phenotype shown here.

Thus, the ethanol-induced enlargement of the liver and the increase of liver triglyceride in *Clock*-mutant mice could be an early marker for alcohol-induced liver failure. By itself, the *Clock*-mutation did produce some modest changes in the levels of some of the genes involved in lipid metabolism. These transcriptional changes did not appear to result in any altered liver function at least with the parameters that we could measure. On the other hand, when we challenged the mutant mice with chronic exposure to ethanol, we found the mutant mice exhibited an increase in liver weight and lipid content. These mutant mice also exhibited ethanol-induced changes in expression of genes involved in triglyceride synthesis and degradation that could explain the effects that we observed. These changes in liver lipid content are similar to those reported in humans with AFL and, thus we propose, that *Clock-*mutant mice could serve as a good model for studying AFL formation.

## Conclusion

We found that ethanol treatment produced a number of changes in the liver of *Clock*-mutant mice without impacting the wild-type controls. First, we found that 8 weeks of exposure to ethanol in the drinking water increased the weight of the liver in *Clock*-mutant mice. Ethanol treatment also increased triglyceride content of liver in *Clock*-mutant and wild-type mice. This increase was larger in the mutant mice. Finally, ethanol treatment altered the expression of a number of genes related to lipid metabolism in the *Clock*-mutant mice.

## Competing interests

The authors declare that they have no competing interests.

## Authors' contributions

TK suggested the primary hypotheses, performed the oil red O staining and real time RT-PCR, and wrote much of the manuscript. TT performed measuring body weight, liver weight, liver TG. SS contributed to the hypotheses and design of the study, and wrote parts of the manuscript. All authors read and approved the final manuscript.
